# Transcriptional Profiling of Hypo- and Hypervirulent *Mycobacterium tuberculosis* Isolates Characterised by Differential Expression of the Moa3 Operon

**DOI:** 10.1007/s00284-026-05038-2

**Published:** 2026-07-27

**Authors:** Jon Mitchell Ambler, Matthys Gerhardus Potgieter, Marisa Klopper, Melanie Grobbelaar, Margaretha De Vos, Samantha Sampson, Robin Mark Warren, Jonathan Blackburn, Nicola Mulder

**Affiliations:** 1 Department of Integrative Biomedical Sciences, Institute of Infectious Disease & Molecular Medicine, Faculty of Health Sciences, University of Cape Town, Western Cape 7925 Cape Town, South Africa; 2 South African Medical Research Council Centre for Tuberculosis Research, Division of Molecular Biology and Human Genetics, Faculty of Medicine and Health Sciences, Stellenbosch University, Cape Town, Western Cape 7602 South Africa; 3 Wellcome Discovery Research Platform for Infection, CIDRI-Africa; and South African Medical Research Council AI for African Population Health Research Unit, University of Cape Town, 7925, Cape Town, South Africa

## Abstract

Despite advances in tuberculosis treatment and prevention, tuberculosis remains a global health threat, responsible for an estimated 1.23 million deaths in 2024 [46], with continued concern over drug-resistant strains. In the Western Cape, South Africa, co-infection of tuberculosis and HIV is a significant public health concern contributing to a higher burden of disease. We compared the transcriptomes of two genetically similar Beijing family strains of *Mycobacterium tuberculosis* under four different growth conditions, including oxidative stress, using three biological replicates for each isolate. We identified an operon containing genes involved in the biosynthesis of molybdenum cofactor that showed consistently lower levels of expression in the hypervirulent isolate. This pathway is known to play a critical role in numerous metabolic and redox processes, and its dysregulation could contribute to the observed differences in disease severity between the two isolates. The *moa* genes, which are involved in the biosynthesis of the molybdenum cofactor, have been previously linked to *M. tuberculosis* virulence through their essential role in redox metabolism and oxidative stress response. Our results support this hypothesis by identifying differential expression of the *moa* operon as a potential mechanism underlying the differences in disease severity between the two isolates. Furthermore, the minimal genetic differences between these isolates make them an ideal system for further study of this pathway and its contributions to tuberculosis pathogenesis.

**Supplementary Information:**

The online version contains supplementary material available at https://doi.org/10.1007/s00284-026-05038-2.

## Introduction

Tuberculosis (TB) is a chronic infectious disease primarily affecting the lungs and caused by *Mycobacterium tuberculosis* (MTB), which can also disseminate to other organs such as the lymph nodes and bones.

Despite improvements in detection and treatment, TB continues to pose a significant disease burden worldwide. The high prevalence of HIV/AIDS in regions such as Africa and Asia has potentiated the impact of TB, leading to greater morbidity and mortality [16]. Those infected with HIV have a significantly greater chance of contracting TB, or for the activation of latent TB, and as both affect the host immune system treatment becomes difficult, particularly in countries with overburdened healthcare systems. According to the 2022 World Health Organisation (WHO) report, the incidence of TB in South Africa is at around 513 people per 100,000 [33], and while this number was declining, the rise of drug resistant TB and disruption of programs due to COVID-19 threatens to rescind this progress. Of particular concern is the incidence of Beijing/W strains frequently associated with drug resistance in this region [17].

The genome of W/Beijing genotype strains differs significantly to that of the H37Rv reference strains [8, 32, 38]. The W/Beijing genotype of MTB has been shown to exhibit large duplications in certain sublineages, which can result in over 300 genes having two copies [10]. Such duplications may have important implications for next-generation sequencing (NGS) experiments that rely on mapping reads to a reference genome, as incorrect gene structure may lead to erroneous inferences regarding the drivers of regulatory differences. Therefore, the choice of reference genome is crucial to obtain an accurate assessment of gene expression. Graph-based references represent a promising solution to this problem, as they enable the integration of closely related reference genomes into the same structure as well-annotated canonical references such as the H37Rv reference genome. By using such tools, researchers can obtain a more comprehensive and accurate understanding of the genomic and transcriptomic variations between different strains of MTB. The differences observed between reference genomes were critical to accurately interpret the expression profiles of the two MTB strains described in this study, particularly with regard to a specific affected operon and a significant regulatory element.

The isolation of two W/Beijing genotype strains in the Western Cape, characterised as hypo- and hyper-virulent based on results from a murine infection model [41], presented a valuable opportunity to investigate the mechanisms underlying the virulence of MTB strains. Despite their close genetic relationship, the examined isolates exhibited minimal genomic variations, none of which appeared to affect known virulence determinants in MTB.

## Materials and Methods

### Mycobacterial Culturing and Sequencing

Culturing of mycobacterial strains was conducted at Stellenbosch University, with the sequencing being done at the Agricultural Research Council in Pretoria. The goal of our study was to identify the genes involved in the altered pathogenicity of the MTB strain SAWC5527 (Hyper-virulent) compared to SAWC507 (Hypo-virulent) [41].

Cultures were grown in Middlebrook 7H9 liquid media with the addition of dextrose and catalase, with growth phases determined by monitoring optical density over time. Samples for RNAseq were extracted during early logarithmic phase (Elog), middle logarithmic phase under control (untreated) conditions (ML(C), where C denotes control), and stationary phase (Stat). A fourth set of samples was harvested during middle logarithmic phase following treatment with 5 mM hydrogen peroxide for 6 hours to simulate growth in the phagosomal environment (ML(T), where T denotes treated). The 5 mM concentration was chosen to match the regime that yielded the largest transcriptional response in *M. tuberculosis* in the survey by Voskuil et al. [43]. Each condition had 3 biological replicates, resulting in 24 total samples sequenced. For the removal of rRNA from the total RNA samples the Truseq stranded mRNA library preparation kit (RS-122–2101) with the Bacterial Ribozero kit (MRZMB126) was used. The samples were sequenced on an Illumina HiSeq 2500 using version 4 SBS chemistry (2x125bp), with approximately 10 million reads per sample loaded into the lane.

### Read Filtering, Alignment, and Differential Expression

Reads were trimmed and filtered using Trim Galore! (version 0.4.0) [21], and aligned to both the H37Rv (accession NC_000962.3) [7] and W-148 (accession NZ_CP012090.1) [12] isolate genomes separately using BWA-MEM [23], and alignments filtered using SAMtools [24]. GenGraph [2] was used to create a graph genome containing H37Rv, H37Ra, and W/Beijing strains to allow mapping of annotations and comparison of gene structure across species. Differential expression analysis was conducted using Cuffdiff (v2.2.1) [42] with a masking gff3 file containing rRNA and tRNA. Cuffdiff uses a Benjamini-Hochberg correction to produce a q-value, and genes with a q-value < 0.05 were classified as significantly differentially expressed.

### Variant Calling

Variant calling was performed using whole genome sequencing reads from SAWC5527 and SAWC507 mapped against both the W-148 and H37Rv reference genomes individually. The pipeline makes use of BWA-MEM [23] for the alignment of the reads, SAMtools [24] for the filtering of the aligned reads, and the GATK HaplotypeCaller [30] for variant calling. The variant files were then filtered using vcftools (0.1.12b) [9] and annotated using SnpEff [6].

## Results

### Genomic Comparison of the Isolates

Alignment of the WGS reads to the W-148 genome produced on average 0.21% more reads than mapping to the H37Rv strain. Variant calling revealed the two isolates differed by 120 variants (SNPs and small indels), summarised by predicted functional impact in Table 1. Of the 85 coding-region variants, 7 were classified as high impact (frameshift or stop mutations), 54 as moderate effect (missense), and 24 as low impact (silent). A per-variant list annotated against the H37Rv reference is provided in Supplementary Table S6. The two isolates shared 4 large deletions relative to the W-148 reference, from 421bp to 2482bp in size.

**Table 1:** Summary of variants detected between SAWC507 and SAWC5527, annotated by SnpEff predicted functional impact (W-148 reference alignment)

SnpEff impact	Effect class	No. variants
High	Frameshift/stop-gain	7
Moderate	Missense	54
Low	Synonymous (silent)	24
Modifier	Upstream/intergenic/other	35
	Total	120

Some notable gene losses include a *esxR* gene, a PE family gene, two universal stress genes, a metalloprotease, a hypoxic response gene, a major facilitator superfamily (MFS) transporter, and an alpha/beta hydrolase. As these large deletions were found in both of the isolates, they were not relevant to the comparison.

### Differential Expression Analysis

**Fig. 1:**
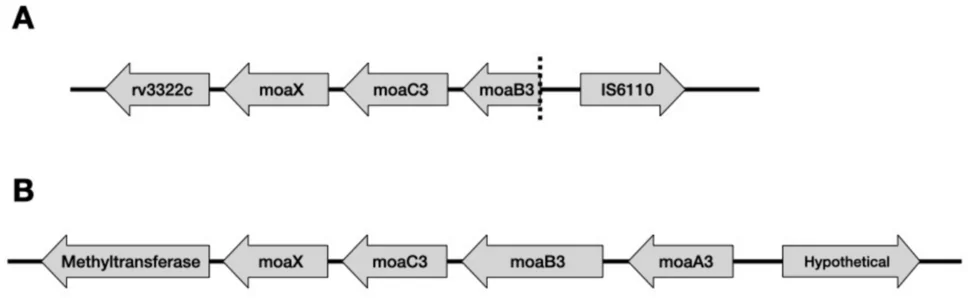
Differing structures of the Moa3 operon. **A** represents the structure in H37Rv while **B** is the structure found in isolate SAWC5537 and SAWC507

**Fig. 2:**
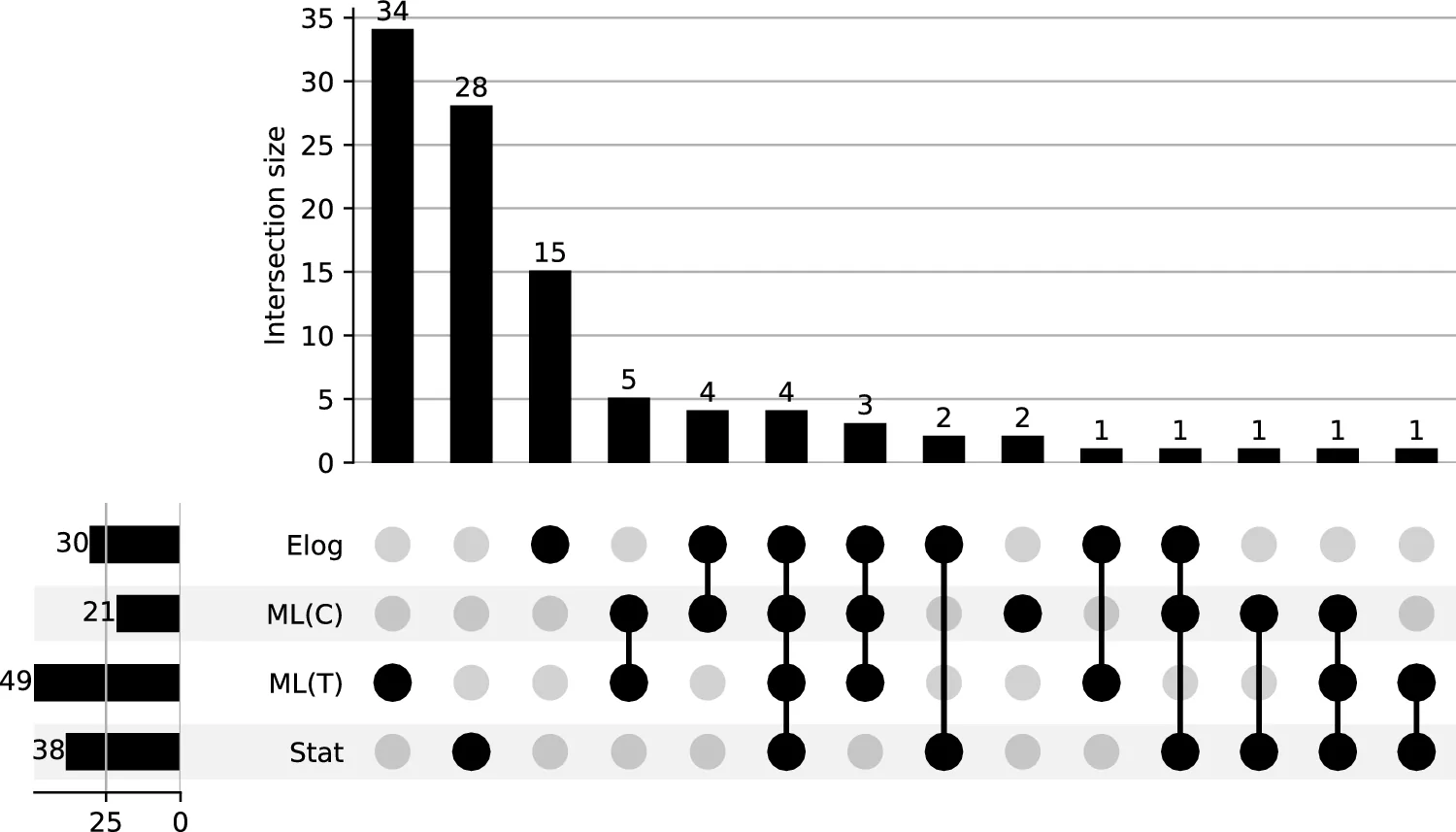
Distribution of differentially expressed genes across growth conditions. UpSet plot showing the size of each intersection of differentially expressed gene sets (*q*-value < 0.05) between the four conditions: early logarithmic phase (Elog), middle logarithmic phase under control (ML(C)) and hydrogen peroxide-treated (ML(T)) conditions, and stationary phase (Stat). Top bars give the size of each intersection; left bars give the total number of differentially expressed genes per condition. Four genes are differentially expressed in all four conditions, while the majority are condition-specific

**Fig. 3:**
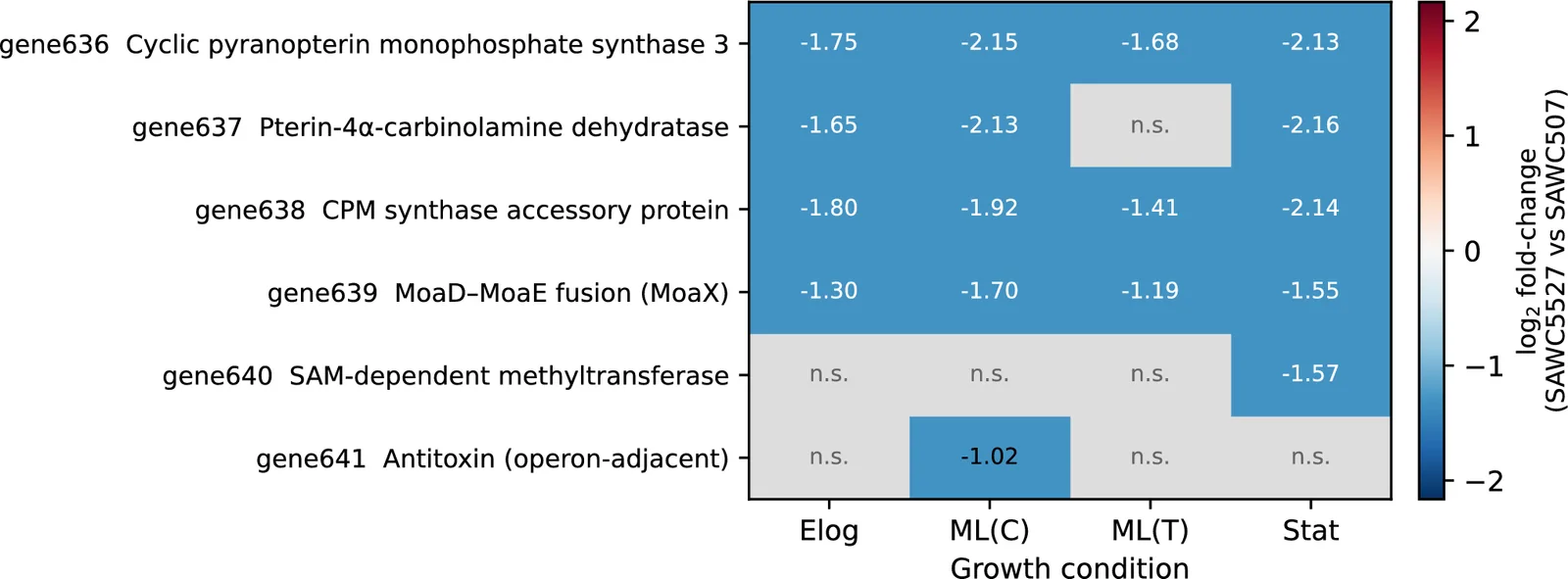
Differential expression of the Moa3 operon in the hypervirulent isolate. Log_2_ fold-change (SAWC5527/SAWC507) for each gene of the Moa3 operon and the adjacent antitoxin gene across the four growth conditions. Cells labelled “n.s.” did not pass the *q* < 0.05 significance threshold in that condition. The four core operon genes (*gene636*–*gene639*) are consistently down-regulated in the hypervirulent isolate across all conditions, with the single exception of *gene637* (TBPG_RS03190) in ML(T)

We identified 102 genes differentially expressed between the isolates across all conditions, with the majority only differentially expressed under a subset of the growth conditions, and 4 found differentially expressed under all 4 conditions (Figs. 1 and 2). When comparing gene expression between the isolates on a per condition basis, the number of differentially expressed genes ranges from 21 to 49 (Table 2). Full lists of the differentially expressed genes are found in the supplementary tables S2, 3, 4 and 5. One of the three operons involved in molybdenum cofactor (MoCo) biosynthesis (the Moa3 operon) was found to have decreased expression in SAWC5527 under all conditions, with $\log_{2}\log_{2}$ fold-changes between *-1.2* and *-2.2* for the core operon genes (Fig. 3); the only gene in this cluster not passing significance in a given condition is TBPG_RS03190 in ML(T). Other MoCo operons and genes were not observed to be differentially expressed, implying that this operon is under independent regulation.

### The Involvement of MoCo in Pathogenesis

MoCo is an important cofactor for enzymes linked to pathogenesis in MTB [29, 44], and aids in survival in the macrophage environment. The horizontal acquisition of the MoCo biosynthesis pathway contributed to *M. tuberculosis* pathoadaptation [22], and the disruption of genes in the hypoxia-responsive operon which includes *moaB1*, *moaC1*, *moaD1* or *moaX* has been shown to impair the ability of MTB to block phagosome maturation, reducing the ability of the bacteria to parasitise macrophages [4, 27, 39]. Some of the MoCo-dependent enzymes include the *narGHI*-encoded nitrate reductase, an important protein for MTB to survive in the oxygen deprived environment of the granuloma and associated with increasing levels of virulence in the pathogen [1, 18]. The observed differential expression of these genes therefore is a key contributor to the difference in virulence between the two isolates.

### Structure of the Moa Operons

MoaR1 is a known regulator of the Moa1 operon [26] but inspection of our WGS data showed a second rearrangement event has split *moaR1* in half in both of the isolates in question (Fig. S1). This appeared to have rendered the Moa1 operon inactive, with low levels of expression under all conditions. The Moa2 operon shows some expression, but none of the genes were differentially expressed under any of the conditions tested, and is under the same transcriptional control as the Moa3 operon. Though ChIP-Seq experiments have been conducted to identify the regulators of MTB genes [15], these were conducted with H37Rv, and due to the altered structure of the Moa3 operon are not applicable.

### The Involvement of the Copper Sensitive Operon (Cso)

**Fig. 4:**
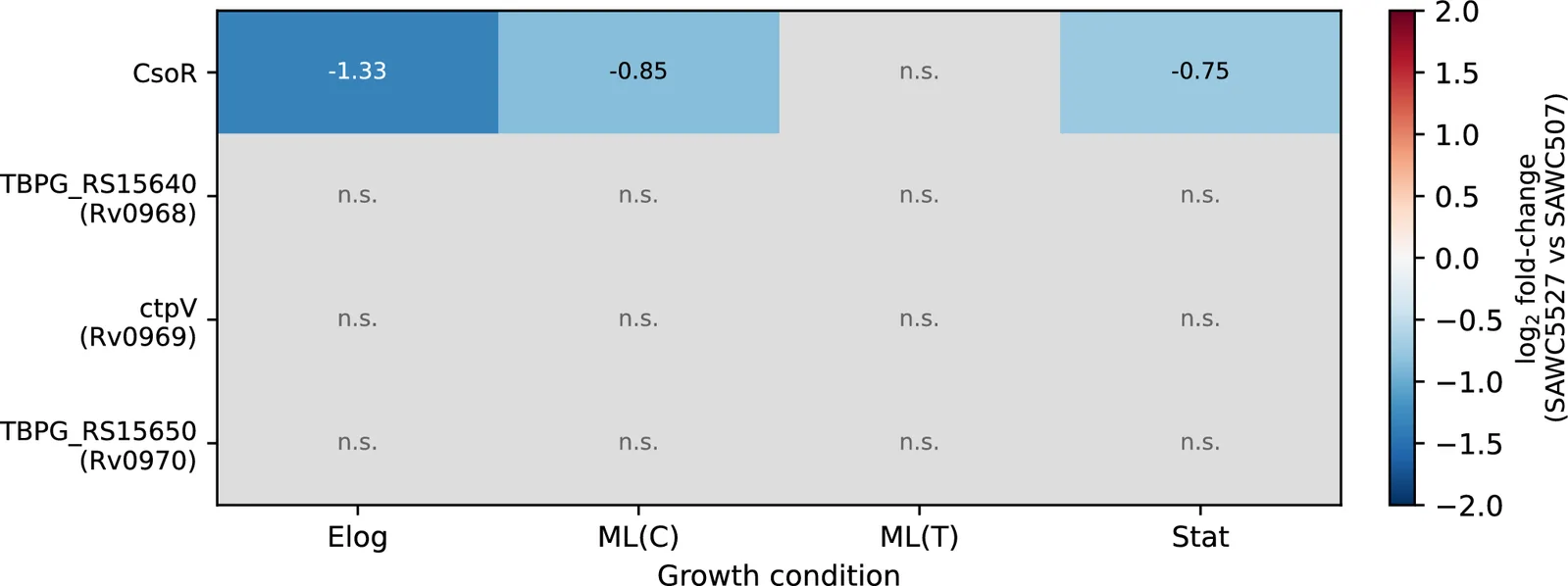
Differential expression of CsoR and the Cso operon. Log_2_ fold-change (SAWC5527/SAWC507) for the copper-sensitive operon repressor CsoR (TBPG_RS15635) and the three structural genes of the Cso operon (TBPG_RS15640/Rv0968, *ctpV*/Rv0969, TBPG_RS15650/Rv0970) across the four growth conditions. Cells labelled “n.s.” did not pass the *q* < 0.05 significance threshold. CsoR is consistently down-regulated in the hypervirulent isolate except under hydrogen peroxide treatment; the structural operon genes do not reach significance in any condition

The copper-sensitive operon repressor CsoR (Rv0967/TBPG_RS15635) was significantly down-regulated in the hypervirulent SAWC5527 strain under early logarithmic $(\log_{2} {\mathrm{FC}} = - 1.33)$, middle logarithmic control $(\log_{2} {\mathrm{FC}} = - 0.85)$, and stationary phase $(\log_{2} {\mathrm{FC}} = - 0.75)$ conditions *(q < 0.05)*; the difference during hydrogen peroxide treatment was in the same direction but did not reach significance (Fig. 4). CsoR is a metalloregulatory repressor induced by copper that is known to regulate the copper sensitive operon (Cso), which contains three genes (*rv0968*–*rv0970*), including *ctpV* (*rv0969*), a metal cation-transporting ATPase/efflux pump. The structural genes of the Cso operon did not show significant differential expression between the two isolates under any condition (Fig. 4). Cu(I) binding to CsoR results in disassociation of the repressor from the operator-promoter region of the Cso operon [25]; the lower CsoR levels in SAWC5527 may therefore reflect reduced copper availability in that isolate rather than a direct effect on operon output.

### Differential Expression of Genes Under Different Growth Conditions

Apart from the genes with an apparent link to MoCo biosynthesis, a number of differentially expressed genes were also noted as potentially contributing to the differences in phenotypes, many only observed under certain conditions. Details of the genes that were significantly differentially expressed with a *q*-value less than 0.05 can be found in the supplementary tables S2, 3, 4 and 5.

#### Early Logarithmic Phase Growth

When comparing the strains during early logarithmic phase growth, we observed differentially expressed genes with proximal variants distinguishing the two isolates. Two examples are TBPG_RS03725 and TBPG_RS15510, with SNPs 54bp and 2102bp upstream respectively. The 54bp variant falls within the likely promoter region of TBPG_RS03725 and could plausibly affect transcription factor binding. The 2102bp variant, by contrast, is unlikely to alter expression directly at this distance unless it coincides with a more distal regulatory element, and is reported here as an observation rather than a proposed mechanism. The first, TBPG_RS03725, is an acetyl-CoA carboxylase that showed a decrease in expression in the more virulent isolate SAWC5527. The second is TBPG_RS15510 (Rv0991c in H37Rv), which is annotated as a FmdB family transcriptional regulator in W-148 that has been identified as one of the genes involved in regulatory mechanisms in response to nitrogen limitation in *Mycobacterium smegmatis* [20, 35]. This gene was also found to have decreased expression in SAWC5527 but is only 333bp long with neither of its flanking genes showing significant differential expression.

#### Middle Logarithmic Phase Growth

During middle logarithmic phase growth, we observed *esxB* (TBPG_RS20410/Rv3874) showing increased levels of expression during both ML(C) and ML(T) conditions in isolate SAWC5527. This secreted virulence factor is required for pathogenesis in *Staphylococcus aureus* and MTB [5]. EsxB is secreted by the ESX-1 system along with EsxA (Rv3875) [14], which did not show any significant changes in expression in any of the observed conditions.

#### Growth Under Treatment with Hydrogen Peroxide

Treatment with hydrogen peroxide yielded the greatest number of differentially expressed genes between the two isolates (49 genes; Table 2), though a large number of these were annotated as hypothetical proteins. Notable genes found in this dataset with links to pathogenicity were a type B diterpene cyclase (TBPG_RS17815) and a diterpene synthase (TBPG_RS17820) that were both found to be significantly down-regulated with half the level of expression in the hyper-virulent strain. Diterpenes have been studied in the past for their potential role in promoting phagolysosome maturation arrest [34, 36] and while it is unclear why the hyper-virulent strain would have lowered expression for these genes, it shows once again that these isolates are responding in different manners to oxidative stress.

Two virulence factors shown to be involved in resistance to oxidative stress are Rv2617c (TBPG_RS12610) and P36 (Exported repetitive protein Erp or Rv3810) [13]. The expression of Rv2617c (TBPG_RS12610) in our dataset was shown to be significantly increased in SAWC5527 during treatment with hydrogen peroxide, while P36 showed an increase but did not pass significance testing.

**Table 2:** Summary of the number of genes found to be differentially expressed between the two isolates under different conditions, with isolate W-148 used as the reference genome

Condition	Total	Up regulated in SAWC5527	Down regulated in SAWC5527
Early log (Elog)	30	23	7
Mid log control (ML(C))	21	16	5
Mid log treated (ML(T))	49	17	32
Stationary (Stat)	38	37	1

P36 is under the control of sigma factor H (SigH) [31] which has lower expression in SAWC5527 relative to SAWC507, though only with a significant q-value during stationary phase growth. SigH has been shown to play a crucial role in the response to environmental conditions including oxidative-stress, envelope damage, hypoxia [28, 37], survival in the phagocytes possible through the modulation of the host’s innate immune response [11], and as a key regulator of the transition phase in *Clostridioides difficile* (formerly *Clostridium difficile*) [40]. The expression of *sigH* also appeared highest during stationary phase, lower during early log phase and lowest in mid log phase growth in both samples possibly indicating a role in dormancy, and that SAWC5527 is generally in a more active state.

#### Stationary Phase

The stationary phase contains the largest set of adjacent differentially expressed genes (7 genes within the TBPG_RS17790–RS17825 region; TBPG_RS17800 was the sole gene in this region not reaching significance, *q = 0.999*) found to be down-regulated in the hyper-virulent strain. These include the previously mentioned diterpene synthesis related genes found in the hydrogen peroxide treated samples. The other genes appear to be likewise involved in metabolism, including trehalose-phosphate phosphatase that is located in the cell wall and induces humoral and cellular immune responses in the host [47]. Though considered to be significantly differentially expressed, many of these genes have substantially lower expression than neighbouring genes in the region: TBPG_RS17795, TBPG_RS17805, TBPG_RS17820, and TBPG_RS17825 had FPKM values of 16–108 in SAWC507 during stationary phase, while TBPG_RS17790 had an intermediate FPKM of approximately 150; these contrast with 262–797 FPKM for the flanking TBPG_RS17785, TBPG_RS17810, and TBPG_RS17815. The non-significant TBPG_RS17800 had a mean of only *∼*28 mapped reads per replicate, consistent with its failure to reach significance. Despite this low absolute expression, inter-replicate variance was small and all seven differentially expressed genes in the region remained statistically significant.

## Discussion

### Drivers of Virulence

Although several genes with potential links to pathogenicity and virulence were observed under specific growth conditions, only two genes - acetyl-CoA carboxylase and FmdB family transcriptional regulator - had a potential driver in the form of a proximal variant. While these genes may contribute to the differential virulence phenotype observed between the two MTB isolates, the most probable primary drivers of the phenotype, based on their consistency and pervasiveness, are the differential expression of the Moa and Cso operons as outlined above.

### Mechanism of Altered Virulence

The high number of differentially expressed genes in the oxidative stress sample and differential expression of genes related to phagosomal maturation arrest (Diterpenes), survival in the macrophage (*sigH*), and resistance to oxidative stress (Rv2617c) point to the differing ability of the two discussed isolates to survive engagement with the macrophage as the key discerning characteristic. The differential expression of the copper-sensitive operon repressor regulated operon Cso supports this narrative, as copper is used in the production of reactive oxygen species in the phagolysosome to destroy internalised bacteria [3, 19] and copper resistance has been shown to be essential for virulence [45].

### Future Steps

In light of the intriguing findings presented in this study, several avenues of further investigation emerge that promise to deepen our understanding of the mechanisms governing tuberculosis pathogenesis. These prospective research directions are essential not only for advancing our knowledge but also for potentially informing future strategies for disease intervention.

#### Identification of Transcription Factors (TFs)

Given the unique gene structure observed in the studied isolates compared to the H37Rv reference strain, it is imperative to unravel the regulatory machinery governing the differential expression of key operons, such as MOA3 and CSO. To this end, comprehensive identification of the specific transcription factors (TFs) responsible for modulating these operons is warranted. Employing techniques such as ChIP-Seq can facilitate the mapping of TF binding sites within the regulatory regions of these operons. Subsequently, a comparative analysis of these binding sites against known TF binding motifs will shed light on the intricate regulatory networks governing molybdenum cofactor biosynthesis and copper resistance. This investigation will provide crucial insights into the transcriptional control mechanisms underlying virulence variations in tuberculosis strains.

#### Functional Characterization of MOA3 and CSO Operons

To gain a deeper understanding of the functional significance of the MOA3 and CSO operons in the context of tuberculosis pathogenesis, it is imperative to conduct detailed experiments aimed at elucidating their roles. One critical aspect to explore is how these operons affect the response and resistance of the isolates to copper, a pivotal element in the host’s defense mechanisms against intracellular pathogens. A series of growth comparisons under varying copper concentrations can provide valuable data on the isolates’ adaptability and susceptibility to this crucial environmental factor. These experiments will not only validate the operons’ involvement in copper resistance but also shed light on the broader regulatory networks governing metal homeostasis in tuberculosis.

#### Elucidating Host-Microbe Interactions

Understanding the interplay between the tuberculosis isolates and host immune cells, especially macrophages, is central to comprehending the observed differences in virulence. Future investigations should delve into the molecular mechanisms underlying the varying abilities of these isolates to survive within macrophages. Specifically, elucidating how the isolates influence phagosomal maturation arrest, survival strategies within the macrophage environment, and resistance to oxidative stress will be of paramount importance. This line of research promises not only to advance our understanding of tuberculosis pathogenesis but also to inform potential therapeutic interventions aimed at enhancing host immune responses.

## Conclusion

In this study, we compared two closely related MTB isolates (SAWC507 and SAWC5527) with different levels of virulence at the genomic and transcriptomic levels. We did not identify any variants within coding regions that could account for the difference in virulence. However, we found that the genes of the Moa3 operon were consistently down-regulated in the hypervirulent SAWC5527 isolate. The acquisition of the *moa* genes is believed to have contributed to the pathoadaptation of MTB, with the Moa3 locus a paralogue of the *moaA1-D1* gene set. We did not observe differential expression of the Moa1 operon or MoaR1, indicating that the Moa3 operon is not regulated by the same regulator as Moa1. This suggests the existence of a second MoCo biosynthetic pathway in MTB strains where the Moa3 operon is not interrupted. This pathway may have eluded detection due to the altered structure of the operon in the canonical H37Rv strain. The Moa3 operon is most likely a downstream expression of an unknown regulatory element or variant, and the altered virulence phenotype may be related to the pathogen’s hypoxia response mechanisms. Although the cause of the differential expression remains unclear, these two isolates provide an ideal system for exploring this new, pathologically relevant pathway. The future steps outlined above represent a roadmap for further investigations that will contribute to our understanding of these mechanisms and may hold the key to more effective strategies for combating this global health threat.

## Supplementary Information

Below is the link to the electronic supplementary material.

Supplementary file 1 (jpeg 600 KB)

Supplementary file 2 (csv 2 KB)

Supplementary file 3 (csv 1 KB)

Supplementary file 4 (csv 3 KB)

Supplementary file 5 (csv 3 KB)

Supplementary file 6 (csv 15 KB)

Supplementary file 7 (docx 15 KB)

## Data Availability

The raw RNA-sequencing data generated during the current study are available in the NCBI Sequence Read Archive (SRA) repository under accession number PRJNA691317 (https://www.ncbi.nlm.nih.gov/sra/PRJNA691317). All other data generated or analyzed during this study are included in this published article and its supplementary information files.

## Code Availability

All tools and software used in this study are referenced and available publicly online at the time of writing.
