# Supplementary figures and images for: Transcriptional Profiling of Hypo- and Hypervirulent Mycobacterium tuberculosis Isolates Characterised by Differential Expression of the Moa3 Operon
Source: Curr Microbiol. 2026 Jul 27;83(9):492. doi: 10.1007/s00284-026-05038-2 (PMC13407613; doi:10.1007/s00284-026-05038-2)

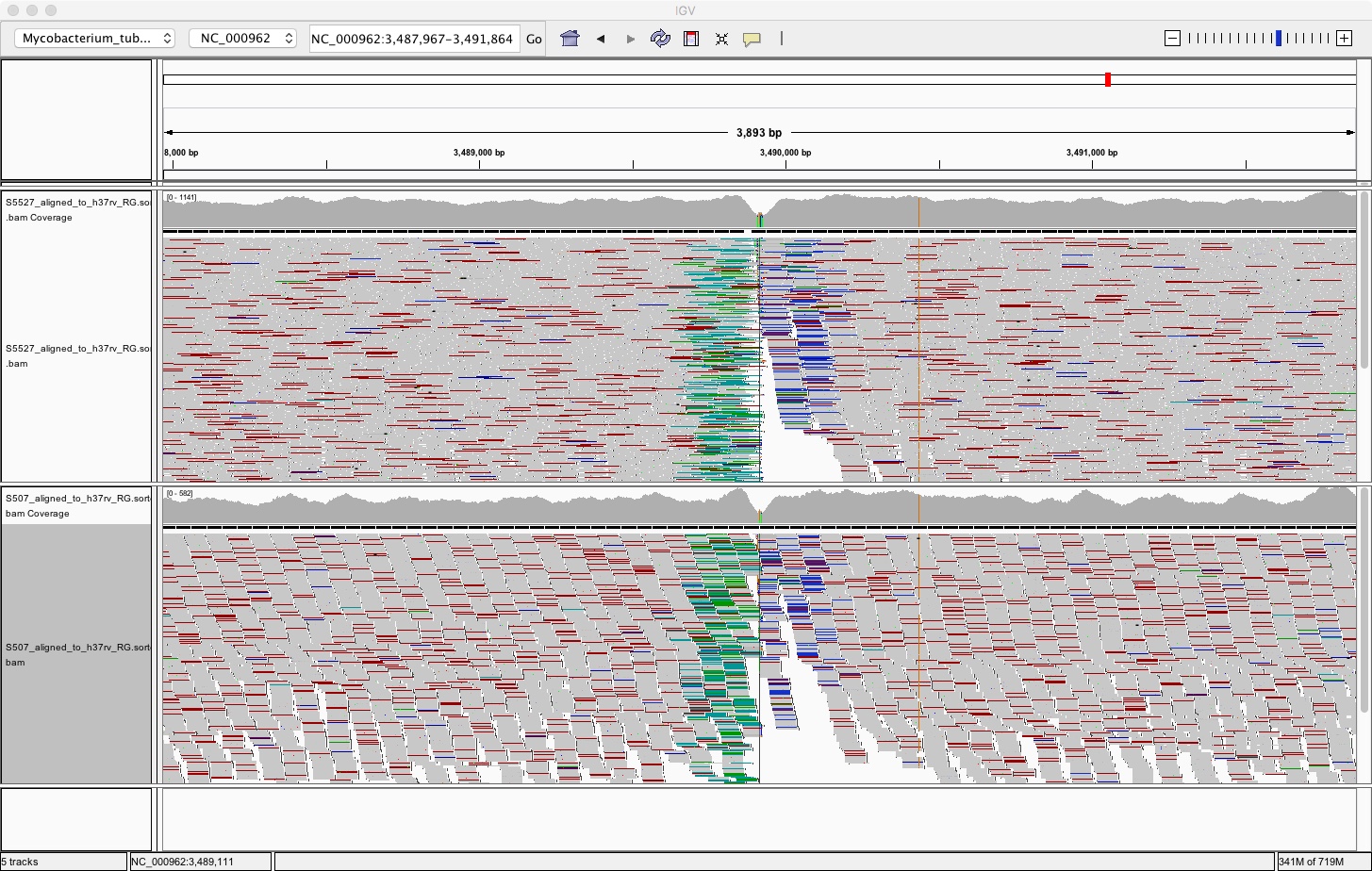

Supplement: Supplementary file 1 — (jpeg 600 KB) [file 284_2026_5038_MOESM1_ESM.jpg]
