# Supplementary material for: Transcriptional Profiling of Hypo- and Hypervirulent Mycobacterium tuberculosis Isolates Characterised by Differential Expression of the Moa3 Operon
Source: Curr Microbiol. 2026 Jul 27;83(9):492. doi: 10.1007/s00284-026-05038-2 (PMC13407613; doi:10.1007/s00284-026-05038-2)
Supplement: Supplementary file 7 — (docx 15 KB) [file 284_2026_5038_MOESM7_ESM.docx]

S1 File.

**Site of chromosomal rearrangement in H37Rv.** This figure shows how reads from isolates SAWC507 and SAWC 5527 appear when aligned to the H37Rv reference genome in the region of the Moa3 operon where a chromosomal breakpoint is found.

S2-S5 File.

**Amalgamated results of the differential expression of genes during the early log phase of growth.** The SNPs column refers to any variants found within the coding sequence of the gene. The operon column high-lights clusters of genes that are all differentially expressed and probably part of an operon. Any gene proximal to the differentially expressed gene that is also differentially expressed is listed. The final column shows the homologue of the gene in H37Rv as predicted by whole genome sequence alignment and annotation overlapping done by the tool GenGraph [[**2**](file:///C:\temp\284_2026_5038_Article.LE.html#CR2)] The purpose of this table is to bring together various pieces of data to identify potential mutational drivers of the differentially expressed gene clusters and provide additional context in the form of homologous genes.

S6 File.

**Per-variant list annotated against the H37Rv reference.** Complete list of the 120 variants detected between SAWC507 and SAWC5527 from the W-148 reference alignment, annotated against the H37Rv reference genome. Columns: Isolate, Position (H37Rv), Ref, Alt, Type, Gene (H37Rv), PE/PPE family (Yes/No), SnpEff impact, Effect, Nucleotide change, Amino acid change. Variants in PE/PPE family genes, which account for approximately 54% of all variants, are flagged separately.
